# Unraveling the Extra Virgin Olive Oil Effect on Inflammation and on Gut and Saliva Microbiota

**DOI:** 10.3390/biom15030338

**Published:** 2025-02-26

**Authors:** Marta Correia, Ana T. P. C. Gomes, Inês Moreira, Jane El Maghariki, Karina Mendes, Maria José Correia, Rui Barros, Joana Cristina Barbosa, Nuno Rosa, Ana Maria Gomes

**Affiliations:** 1 CBQF-Centro de Biotecnologia e Química Fina—Laboratório Associado, Escola Superior de Biotecnologia, Universidade Católica Portuguesa, Rua Diogo Botelho 1327, 4169-005 Porto, Portugal; mmcorreia@ucp.pt (M.C.); inesdcmoreira@gmail.com (I.M.); janemaghariky@gmail.com (J.E.M.); rbarros@ucp.pt (R.B.); jcbarbosa@ucp.pt (J.C.B.); 2 Centro de Investigação Interdisciplinar em Saúde (CIIS), Faculdade de Medicina Dentária, Universidade Católica Portuguesa, Estrada da Circunvalação, 3504-505 Viseu, Portugal; apgomes@ucp.pt (A.T.P.C.G.); kmendes@ucp.pt (K.M.); mcorreia@ucp.pt (M.J.C.); nrosa@ucp.pt (N.R.)

**Keywords:** EVOO, glycated haemoglobin, inflammatory biomarkers, microbiota, prebiotic

## Abstract

Extra virgin olive oil (EVOO) with a high content of polyphenols has attracted attention due to its proved beneficial effects in decreasing the risk of cardiovascular disease, modulating cholesterol levels (HDL and LDL), modulating inflammatory markers, and decreasing the levels of haemoglobin1Ac, suggesting that EVOO can have an impact in glycemia regulation. This study assessed the impact of the consumption of a northern Portuguese polyphenol-rich EVOO with a high profile of bioactive molecules on several parameters, such as saliva and serum inflammatory biomarkers, and explored EVOO impact on gut and oral microbiota regarding Bacillota and Bacteroidota content. Thus, the impact on glycated haemoglobin (HbA1c), low-density lipoprotein cholesterol (LDL-C), C-reactive protein (CRP), inflammatory biomarkers, and faecal and salivary microbiomes were evaluated before and after the exposure to EVOO. The results showed that EVOO promotes a decrease in the levels of HbA1C and in the pro-inflammatory interleukin IL-1β, associated with inflammatory processes. Moreover, EVOO intake modulated gut and oral microbiota, increasing Bacteroidota in both ecological niches and Bacillota in the oral microbiota, both phyla being associated with health, demonstrating a prebiotic effect.

## 1. Introduction

Extra virgin olive oil (EVOO), particularly high-polyphenol EVOO, is known for decreasing the risk of cardiovascular disease, modulating age-related HDL cholesterol levels, decreasing LDL cholesterol levels, and improving oxidative-stress-related outcomes [1]. Indeed, EVOO’s phenolic and lipid profiles have been extensively studied in clinical trials by numerous authors for their beneficial health attributes [2,3]. Portugal is known for its high-quality EVOO and its great variety of olive tree cultivars, one of which is the monovarietal Santulhana EVOO, produced in the northern region of Trás-os-Montes in the municipalities of Bragança, Vimioso, and north of Macedo de Cavaleiros. This cultivar is productive, presenting an oil yield of around 20%, and well adapted to cold climates and high-altitude areas. In the scope of a previous project (“Bio-n2-value”, NORTE-01-0145-FEDER-000030), it was demonstrated that similar monovarietal EVOOs from different locations in the Trás-os-Montes region in the north of Portugal revealed different lipid and polyphenolic compositions at concentrations that may support EVOO’s biological functionality [4].

This specific northern Portuguese polyphenol-rich EVOO, besides being a hallmark of the Mediterranean diet (MedD), shows distinctive characteristics, studied and published elsewhere by our group [5]. Indeed, it has been shown that this high phenolic-EVOO (particularly in hydroxytyrosol, tyrosol, and oleuropein) positively affects metabolic parameters and cardiovascular disease risk factors, as well as a significant decrease in the glycation of haemoglobon1Ac [5], which also suggests a role for EVOO in glycemia regulation [6].

EVOO phenolic compounds, such as oleuropein, tyrosol, and hydroxytyrosol, are well known for their beneficial effects on increasing longevity and as antioxidants and anti-inflammatory agents [7]. In particular, hydroxytyrosol is recognised to hold significant antioxidant action, inflammation modulation, and autophagy inhibition, biological properties that might explain EVOO’s reported contribution towards healthy aging and in delaying the development and progression of chronic diseases [6]. EVOO’s anti-inflammatory and antioxidative properties have also been shown to prevent muscle mass and function loss, decreasing the risk of sarcopenia amongst adults and the elderly [8]. Chronic immune-mediated inflammatory diseases (IMID), such as rheumatoid arthritis, inflammatory bowel disease (IBD), and multiple sclerosis, among others, have been found to benefit from EVOO due to its ability to modulate the associated inflammatory profiles [9,10]. For instance, in vitro and IBD animal studies provide solid evidence on the mechanisms by which EVOO, specifically its components oleuropein and hydroxytyrosol, exert their antioxidative, anti-inflammatory, immunomodulatory, and anti-tumor effects [11]. This accumulated evidence supports the use of EVOO as an excellent dietary intervention to alleviate symptoms and aid in the management of inflammatory conditions, such as IBD [11]. Moreover, it is well established that chronic low-grade inflammation levels are implicated in the pathogenesis of several chronic non-communicable diseases [12], and pro-inflammatory markers, including interleukin-6 (IL-6), IL-18, IF-gamma, and tumor necrosis factor-α (TNF-α) have been prospectively associated with the progression of these diseases [13].

It is becoming more and more apparent that research looking for associations between diet and its anti-inflammatory role, including several large-scale dietary interventions, should use approaches based on the overall diet quality rather than focusing on a single nutrient [14]. Interestingly, diet modulates inflammation, and higher diet quality has been associated with favorable inflammation profiles [15,16]. In this context, the Dietary Inflammatory Index (DII) was developed to provide a quantitative means for assessing the role of diet in relation to health outcomes ranging from the blood concentrations of inflammatory cytokines to chronic diseases and was designed to be universally applicable across all human studies with adequate dietary assessment [17]. It is formed by an a priori literature-based method and is based on 45 food parameters including individual nutrients (e.g., omega-3 fatty acids), compounds (e.g., flavonoids), and food items (e.g., garlic, ginger) that were identified within the literature as possessing either anti- or pro-inflammatory properties. The DII has now been validated in 29 studies with a range of inflammatory markers, including CRP, IL-6, and TNF-α [18]. A strategic advantage of the DII is that, in contrast to individual dietary compounds, the investigation of dietary patterns acknowledges the food matrix or the complex interactions of nutrients and compounds within foods and dietary patterns.

Since the development of the current DII in 2014, over 450 studies have investigated the association between the DII and a diverse range of chronic disease-related outcomes. The DII has been robustly associated with an increased risk of several inflammation-related diseases, including diabetes [19], cardiovascular disease (CVD) [20], and colorectal cancer [21], with high levels of DII reflecting a more inflammatory diet and consequently being associated with disease incidence and mortality [22]. However, some studies did not find an association between DII scores and a higher risk of CVD, ischemic heart disease, cerebrovascular disease, and others [23].

Importantly, EVOO has also been shown to alter gut microbiota due to its composition of phenolic compounds potentially modifying the oxidative status of the host’s intestinal barrier, inflammation, and immune response [24,25]. Nonetheless, literature is not consistent, as some studies lack clarity on whether the observed health benefits arise from EVOO’s properties or because of a dietary model, such as MedD [26]. Additionally, the human oral cavity hosts the second most plentiful and diverse microbiota after the gastrointestinal tract. Currently, the human oral microbiome present in each individual at a particular time point is believed to consist of more than 250 species and several studies have shown that the microbiome plays a crucial role in human health and disease [27]. Knowing this, there is a growing interest in targeted interventions that can optimise its function and its action in reducing inflammatory mediators [28]. Several nutrients are implicated in modulating pro-inflammatory and anti-inflammatory cascades, influencing baseline inflammatory status [29]. In recent decades, there has been a shift to a “westernized” diet, which has staple foods, such as farmed animal meats, dairy products high in sugar, refined vegetable oils, and processed grains. These products interact with the oral microbiota and exert a selective pressure, favoring the survival and replication of acid-producing and acid-tolerant organisms and leading to pathological changes in the oral microbiota [30]. Interestingly, both the gut microbiota and the oral microbiota are physically connected, and exciting evidence shows complex and important connections between them. The interplay of the two microbiomes may contribute to the pathological processes of many diseases (diabetes, rheumatoid arthritis, nonalcoholic fatty liver disease, inflammatory bowel disease, cancers). Oral bacteria may be internalised either through the enteral route, the hematogenous route, or the immune cell migration route. Interestingly, salivary pathogens such as *Fusobacterium nucleatum* and *Aggregatibacter actinomycetemcomitans* are also reported to affect the gut microbiota [31].

Therefore, this study aims to evaluate the impact of a particular northern Portuguese polyphenol-rich EVOO with a high profile of bioactive molecules on saliva and serum inflammatory biomarkers and explore EVOO’s impact on gut and oral microbiota, regarding the relationship between *Bacillota* and *Bacteroidota*.

## 2. Materials and Methods

### 2.1. Data Collection

All variables and biological samples came from a study with methodology and details fully described elsewhere, with ethical approval received from the Ethical Committee Board for Health of Universidade Católica Portuguesa (Project nº 171-CES/UCP) and registered on the ClinicalTrials.gov platform under NCT05852275 [5]. Briefly, samples used in this study came from a convenience sample of 37 individuals receiving 30 mL of northern Portuguese EVOO daily for 100 consecutive days. This specific EVOO was assessed for both chemical and biological properties and revealed high levels of polyphenols (224.9 µg GAE/g), known for their significant antioxidant effects (235.49 ± 4.42 µmol of Trolox equivalents/mL), as well as a rich profile of monounsaturated fatty acids, particularly oleic acid (>70%) meeting the EFSA health claim requirements (5 mg of hydroxytyrosol, oleuropein, and tyrosol per 20 g of EVOO) and contributing to the protection of blood lipids from oxidation through the activity of polyphenols [32,33,34]. Variables were analysed prior to EVOO exposure and a final assessment after EVOO exposure. Serum biomarkers were analysed using a capillary blood sample obtained from a finger and measured using the Cobas^®^ b101 device (Roche, Basel, Switzerland), which enables the rapid quantitative assessment of glycated haemoglobin (HbA1c) and low-density lipoprotein cholesterol (LDL-C).

This study also collected information regarding dietary habits using PREDIMED and, regarding physical exercise, IPAQ (International Physical Activity Questionnaire—Short Form) questionnaires. Stool and saliva samples were collected before and after the consumption of EVOO and were stored at −80 °C until further analyses. Importantly, all participants, to be enrolled, consumed olive oil but not extra virgin olive oil, therefore excluding from their diets the EVOO typically rich in polyphenolic compounds (oleocanthal, oleuropein, and hydroxytyrosol) until the beginning of the study. Additionally, 3-day dietary logs were collected from every participant, making the dietary comparison possible.

### 2.2. Saliva Sample Collection, Packaging, and Storage

Unstimulated whole saliva (UWS) samples were collected by spitting, according to previously established procedures [32], and kept refrigerated until transportation. Due to their potential biological risk, saliva samples were manipulated in a Level II safety laboratory. The UWS samples were aliquoted into several microtubes, depending on the volume initially collected, and were immediately stored at −20 °C until further analysis.

### 2.3. Salivary Inflammatory Protein Quantification

Salivary inflammatory proteins were quantified through Multiplex Immunoassay technology using a commercial Multiplex^®^ Map Human High Sensitivity T Cell Magnetic Bead Panel Kit (Millipore, Burlington, MA, USA) customized with magnetic microspheres coated with specific antibodies for anti-inflammatory proteins (INFγ and IL-4) and pro-inflammatory proteins (TNFα and IL-1β). Thus, the saliva samples were centrifuged for 10 min at 10,000 rpm and 4 °C, and the supernatant was used for Multiplex quantification according to the manufacturer and analysed in a Bio-Plex^®^ MAGPIX™ Multiplex Reader (Bio-Rad, Berkeley, CA, USA).

### 2.4. Faecal Microbiome Profile

Microbial DNA from faecal samples was extracted using an optimised procedure through the application of the NZY tissue genomic DNA (gDNA) isolation kit (NZYtech, Lisboa, Portugal) according to the manufacturer’s instructions, with some modifications. The procedure began with approximately 250 mg of the sample in 1.5 mL centrifuge tubes. First, the samples underwent a washing step where the pellet was resuspended in 1 mL of TE buffer (1X). The samples were then vortexed vigorously and centrifuged (20,000× *g*, 3 min). After discarding the supernatant, 1000 µL of lysozyme (10 mg/mL in TE 1X) was added, mixed, and vortexed and the mixture was incubated at 37 °C for 2 h. Upon centrifugation (20,000× *g*, 3 min), the resulting pellet was resuspended in 1000 µL of NT1 buffer, and 200 µL of this mixture was transferred to a new Eppendorf, to which 25 µL of Proteinase K solution was added; the resulting mixture was incubated at 56 °C for 2 h, with occasional vortexing. Following the pre-lysis step, the sample lysis took place by adding 200 µL of the NL buffer and ethanol (100%, 210 µL). Adding ethanol and binding DNA were performed for each sample at a time. After discarding the flow-through, the silica membrane washing steps were performed. Centrifugation (13,000× *g*, 2 min) and flow-through discard were performed after every buffer addition. The washing step was performed twice using 600 µL of NW2 buffer to isolate bacterial DNA from the stools. Once again, the sample was centrifuged (13,000× *g*, 2 min) to dry the silica membrane. Finally, the elution step was performed, with the addition of 100 µL of buffer NE (preheated to 70 °C) to the silica column, incubation for 1 min at room temperature, and centrifugation (13,000× *g*, 2 min), and the resulting filtrate was collected to quantify the eluted DNA from the silica column. The DNA samples (25 µL), isolated from human stools, were subjected to Illumina metagenomic sequencing analysis. Samples underwent a procedure of quality control at every step for better accuracy and reliability of sequencing data. The primers for region 16S V3–V4 were used, and the amplicon sequence variant (ASV)-based analysis was selected.

### 2.5. Salivary Microbiome Profile

Bacterial DNA was extracted from saliva samples using the KingFisher magnetic particle processor (Thermo Electron, Vantaa, Finland) with a MagMAX Microbiome Ultra Nucleic Acid Isolation Kit according to the manufacturer’s instructions. Briefly, 400 µL of the samples was transferred to 800 µL of lysis buffer to the bead tubes. Two successive rounds of 30 s bead beating were performed using a 4-Place Mini Bead Mill Homogeniser (VWR^®^, Radnor, PA, USA) and centrifuged for 2 min at 14,000× *g*. Extraction was performed following the kit manufacturer’s instructions for bacterial DNA extraction involving proteinase K treatment and subsequent purification by using the KingFisher device (Melbourne, Australia). The KingFisher protocol included the binding of nucleic acids on magnetic beads, five washing steps, and elution in a 12-well strip. In the end, DNA was quantified using the μDrop Plate (Thermo Fisher Scientific, Waltham, MA, USA) and stored at −20 °C until used in the bacterial quantification experiments.

Quantification of total bacterial load and the bacterial phyla *Bacillota* and *Bacteroidota* was performed by quantitative real-time PCR (qRT-PCR) using universal primers targeting bacterial 16S rRNA gene (926F: 5′ AAACTCAAAKGAATTGACGG 3′; 1062R: 5′ CTCACRRCACGAGCTGAC 3′) and specific primers for *Bacillota* (928F-firm: 5′ TGAAACTYAAGGAATTGACG 3′; 1040FirmR: 5′ACCATGCACCACCTGTC 3′) and *Bacteroidota* (798cfbF: 5′ CRAACAGGATTAGATACCCT 3′; cfb967R: 5′ GGTAAGGTTCCTCGCGCTAT 3′). Primer sequences were taken from Bacchetti De Gregoris et al., 2011 [33]. Each DNA sample was analysed in duplicate in a PCR reaction of a total volume of 10 μL using the NZYSpeedy qPCR Green Master Mix (2x) (MB224, NZYtech, Lisboa, Portugal) with 0.4 μM of each forward and reverse primers and 1ng DNA for each reaction. Amplification and detection of DNA by real-time PCR were conducted with the CFX96 Touch Real-Time PCR Detection System (Bio-Rad, Berkeley, CA, USA) according to the following PCR conditions: 95 °C for 3 min and 40 cycles of 95 °C for 5 s and 61.5 °C for 20 s. To perform absolute quantification of total bacterial load (16S rRNA gene) and *Bacillota* and *Bacteroidota* by qRT-PCR, standards for these bacterial assays were prepared according to the following protocol. The 16S rRNA gene sequences of *Staphylococcus capitis* and *Porphyromonas gingivalis* were amplified using the primers mentioned above and the PCR products were purified using the NZYGelpure kit (MB01101, NZYtech). Purified DNA fragments were cloned in the pNZY28 vector, followed by transformation into *E. coli* Competent Cells (MB05301, NZYtech). Plasmid DNA was purified using the NZYMiniprep kit (MB01001, NZYtech) and quantified using the μDrop Plate (Thermo Fisher Scientific). To generate a standard curve for absolute qRT-PCR quantification, plasmid DNA containing a 16S rRNA gene sequence of Staphylococcus capitis was used as the standard for total bacteria and *Bacillota* assays, while the plasmid containing 16S rRNA gene sequence of *Porphyromonas gingivalis* was used as the standard for *Bacteroidota* assays. Duplicate tenfold dilutions of plasmid DNA corresponding to seven nonzero standard concentrations were used and ranged from 2.77 × 10^10^ to 2.77 × 10^4^ copies of DNA per *reaction* for 16S rRNA, 1.44 × 10^8^ to 1.44 × 10^2^ for *Bacteroidota*, and 1.74 × 10^9^ to 1.74 × 10^3^ for *Bacillota*. The DNA copy numbers of the 16S rRNA gene for *Bacillota* and *Bacteroidetes* within each sample were calculated from the standard curve. For copy number correction, the DNA copy number of each bacterial phylum was divided by the DNA copy number of the 16S rRNA gene.

### 2.6. Dietary Data Collection and Dietary Inflammatory Index (DII)^®^ Calculation

DII is an algorithm that scores diet according to its inflammatory potential [34]. Each DII component was compared to the standard global as a Z-score, which was achieved by subtracting the standard mean from the amount reported and dividing this value by its standard deviation. Then, this value was converted to a centred percentile score. To achieve a symmetrical distribution with values centred on 0 (null) and bounded between −1 (maximally anti-inflammatory) and +1 (maximally pro-inflammatory), each percentile score was doubled and then ‘1’ was subtracted [35]. Higher DII scores indicate that the diet has a higher pro-inflammatory effect, and lower DII scores represent a more anti-inflammatory diet. Thirty-seven dietary components and food products were used to calculate the DII score, including 29 anti-inflammatory elements, namely monounsaturated fatty acids; polyunsaturated fatty acids; n-3 fatty acids; n-6 fatty acids; fiber; alcohol; vitamins A, D, E, C, and B6; carotene; thiamine; riboflavin; niacin; folic acid; magnesium; selenium; zinc; flavan-3-ol; flavones; flavonols; flavonones; anthocyanidins; isoflavones; caffeine; garlic; onion; green/black tea; and eight pro-inflammatory elements, namely carbohydrates, protein, total fat, saturated fatty acids, trans fat, cholesterol, iron, and vitamin B12 [34].

### 2.7. Statistical Analysis

Statistical analysis involved importing dietary intake data into SPSS version 23, applying log transformations to normalise skewed variables, and utilising parametric tests (such as *t*-tests) to compare the mean levels of biochemical, metabolic, inflammatory, microbiota, and dietary data comparing EVOO’s effect. Multiple linear regression and logistic regression were used to adjust for physical activity, energy intake, age, and sex when examining BMI and waist circumference. Habitual intake was estimated using detailed 24 h recall for three consecutive days, and the mean level was calculated. Statistical significance was determined at *p* < 0.05 using appropriate tests, including the chi-square test, *t*-test, or non-parametric tests. Spearman nonparametric correlations were employed to assess relationships between variables.

## 3. Results

### 3.1. Characteristics of the Individuals

Variables were collected from 33 individuals (eight men and 25 women), aged between 19 and 55 (average 33.5 ± 11.2 years). No significant changes were observed in participants’ physical activity, comparing the beginning and the end of the study. In our previous publication, we showed no significant changes regarding anthropometry and body weight, although there was a tendency toward a lower percentage of total fat mass in women at the end of the study [5].

Additionally, it was shown that HbA1c (5.12 ± 0.32; 4.93 ± 0.24, *p* = 0.000) and LDL-c (92.63 ± 24.53; 83.23 ± 27.63, *p* = 0.024) significantly decreased with EVOO but no significant changes (*p* > 0.05) in the profile of the consumption of fatty acids, lipids, or cholesterol were observed (Table 1). All the other parameters did not show any significant changes between the beginning and the end of this study.

### 3.2. EVOO’s Effect on Metabolism and Inflammation

**Figure 1:**
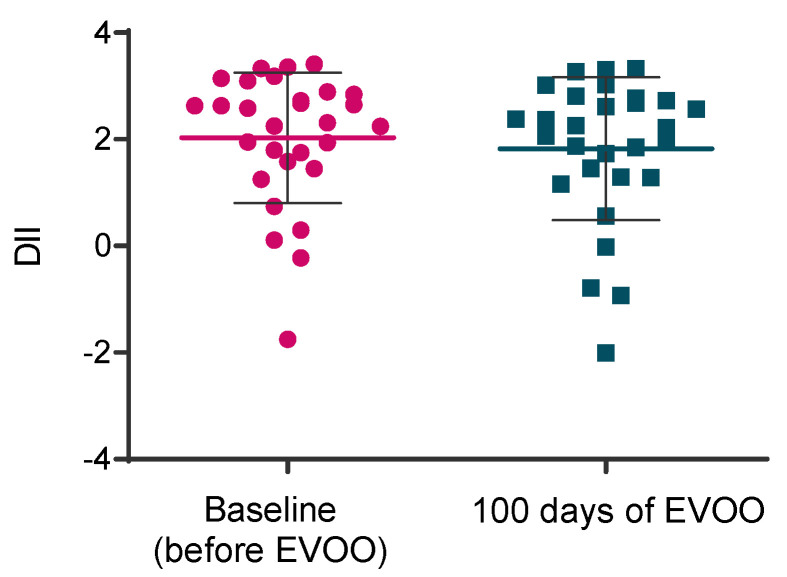
Participants’ Dietary Inflammatory Index before and after EVOO intake.

It was observed that there were no differences in the participants’ diet’s impact regarding its inflammatory potential on the DII before and after EVOO intake (2.178 ± 0.97; 1.93 ± 1.21, *p* = 0.335) (Figure 1). Hence, any metabolic, inflammatory or microbiome variation should be attributable to the EVOO intervention since the participants not only maintained their habitual diets before and after the intervention but also because diets were not significantly different amongst participants.

**Figure 2:**
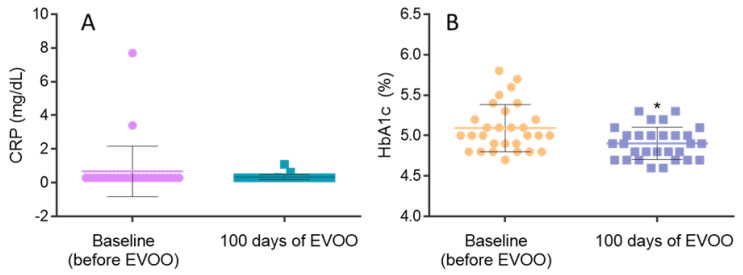
Serum analytical parameters evaluated before and after EVOO exposure. (**A**) CRP quantification and (**B**) HbA1c quantification (* (*p* < 0.05) significantly different from HbA1c at baseline).

Serum biomarkers related to metabolism and inflammation, such as CRP and HbA1c, were evaluated before and after 100 days of EVOO consumption. The results showed no significant changes attained for CRP levels (Figure 2A). Other than the absence of statistical significance, it is important to highlight that CRP concentration from two participants of this study suffered a decrease of 3.10 and 7.40 mg/dL after EVOO exposure. This participant had contracted COVID-19 prior to the initial assessment, which may account for the value of 7.40 mg/dl and its subsequent decrease. Interestingly, the two participants whose CRP levels decreased after EVOO consumption had an increase in the DII score. Concerning HbA1c levels, the results showed a decrease of glycated haemoglobin of 0.10% (*p* = 0.0393) promoted by the intake of EVOO (Figure 2B).

**Figure 3:**
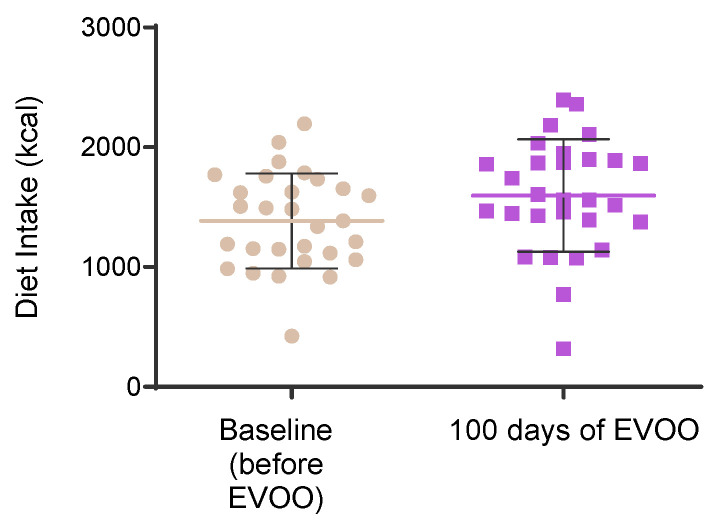
Dietary intake in calories before and after EVOO exposure.

Dietary intake was also evaluated at baseline and after 100 days of EVOO consumption and no significant differences were observed for daily calorie intake (Figure 3). 

### 3.3. Salivary Inflammatory Proteins Quantification

**Figure 4:**
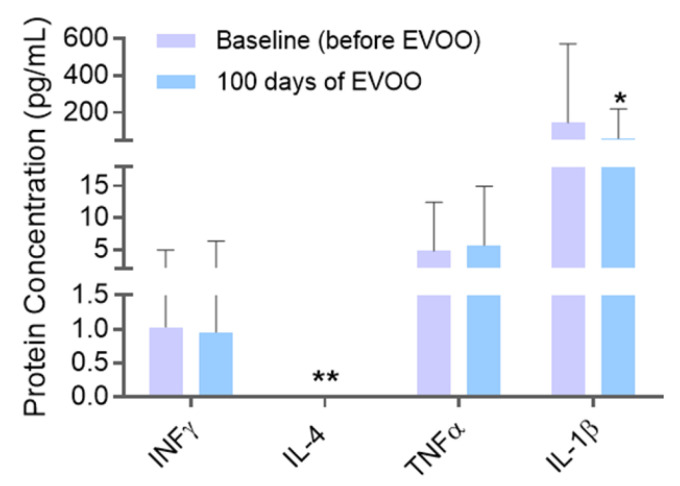
Salivary pro- and anti-inflammatory protein quantification before and after EVOO consumption. * (*p* < 0.05) compared to baseline (before EVOO). ** IL-4 not detected in samples.

Multiplex Immunoassay technology allowed for the simultaneous quantification of pro- and anti-inflammatory proteins present in participants’ saliva samples (Figure 4). Anti-inflammatory proteins INFy and IL-4 did not experience any changes from basal levels after taking EVOO. The same results were achieved for pro-inflammatory TNFα. However, a distinct profile was found for pro-inflammatory IL-1β. After EVOO consumption, this interleukin suffered a statistically significant decrease of 87.14 pg/mL (ANOVA, *p* < 0.05).

### 3.4. Faecal and Salivary Microbiome Profiles

**Figure 5:**
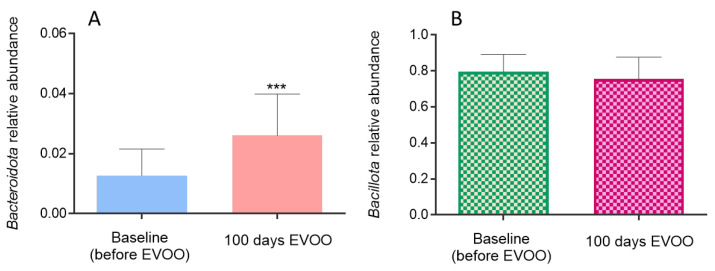
Relative abundance of *Bacteroidota* (**A**) and *Bacillota* (**B**) in stool samples. *** (*p* < 0.001) compared to relative abundance at baseline (before EVOO).

The quantification of the *Bacillota* and *Bacteroidota* was performed in stool and saliva samples. Although no significant differences were achieved in the relative abundance of Bacillota in stool samples (Figure 5B), *Bacteroidota* content suffered a significant increase after EVOO consumption (*p* = 0.0001) (Figure 5A).

**Figure 6:**
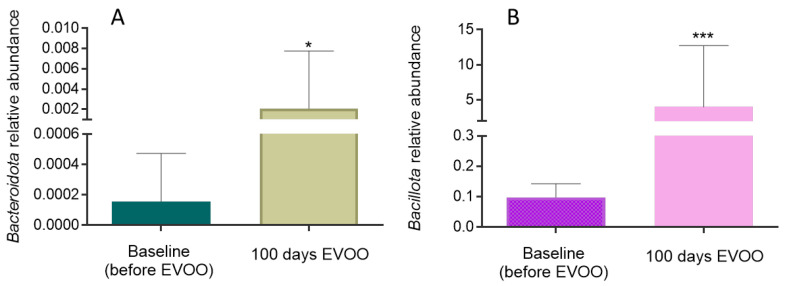
Relative abundance of *Bacteroidota* (**A**) and *Bacillota* (**B**) in saliva samples. * (*p* < 0.05), *** (*p* < 0.001) compared to relative abundance at baseline (before EVOO).

Figure 6 shows the results achieved in the relative abundance of *Bacteroidota* (A) and *Bacillota* (B) in saliva samples from 17 patients before and after EVOO consumption. In both cases, an increase in the relative abundance of *Bacteroidota* (*p* = 0.0456) and *Bacillota* (*p* = 0.0018) after 100 days of EVOO consumption is observed.

## 4. Discussion

The benefits of MedDiet are undeniable in promoting health, being associated with lower risk of mortality, cardiovascular and metabolic diseases, and cancer [36,37]. One of the components of this diet is extra virgin olive oil (EVOO), whose benefits are related not only to the high levels of monounsaturated fatty acids but also to the content in phenolic compounds [38,39,40]. These compounds are responsible for EVOO oxidative stability and sensorial attributes and are known to exert antioxidant, anti-inflammatory, insulin-sensitising, cardioprotective, antiatherogenic, neuroprotective, immunomodulatory, and anticancer activities [38,41]. In fact, there are multiple studies in the literature attesting EVOO’s capacity to modulate chronic immune-mediated inflammatory diseases, such as rheumatoid arthritis and inflammatory bowel disease, among others [38,41]. Additionally, there is also evidence that links EVOO to a significant reduction of cardiovascular events, better glycemia control management, and promotion of intestinal health since it stimulates a higher biodiversity of beneficial gut bacteria [5,38].

Because diet consistently shows the ability to regulate inflammation, this study used the DII to monitor the diet’s associated inflammation potential. Indeed, the DII’s usefulness (apart from common serum biomarkers) translates to direct association between dietary exposures and clinical events as well as being a non-invasive tool [42]. Importantly, the inverse association between the DII and adherence to the Mediterranean diet shown in the literature is associated with decreased levels of inflammatory biomarkers (including CRP) [43]. In this study, the DII did not differ throughout the intervention period, meaning that diet cannot be accountable for the inflammatory and microbiota changes in the gut and saliva. Indeed, this work reports the effect of EVOO consumption on some inflammatory biomarkers, such as CRP, salivary anti- and pro- inflammatory proteins, and metabolic markers, such as HbA1c. The results showed a direct relationship between the decrease of HbA1c and IL-1β after EVOO intake. This pro-inflammatory cytokine has been associated with β-cell damage with several regulatory functions in inflammatory responses and metabolism; it can regulate insulin secretion and promote β-cell apoptosis which can eventually lead to T2DM [43]. In fact, some studies refer to this association between HbA1c and IL-1β levels in T2DM patients, showing a decrease in their levels after treatments [44].

As an inflammation biomarker, HbA1c has also been associated with the CVD risk factor [5]. In cardiovascular disorders, the production of adhesion molecules that cause vascular lesions is simulated by inflammatory cytokines, such as IL-1β and TNF-α. It is expected that in healthy individuals, a suppression in the synthesis of these molecules leads to the reduction of inflammatory processes, diminishing cardiovascular events [45,46]. Despite the absence of changes in TNFα concentration, the decrease in the IL-1β levels after EVOO attests, once again, to the beneficial effects of EVOO in preventing CVD [47]. Interestingly, agents that block IL-1β activity like IL-1β antibodies and the IL-1 receptor antagonist have been described as improving inflammatory diseases such as T2DM, arthritis, gout, and heart failure [48]. Also, other agents with anti-inflammatory properties against IL-1β, including plant-derived substances such as resveratrol and curcumin, well-known polyphenols, have been shown to reduce IL-1β [49] and bone loss in animal models of experimental periodontitis [50,51]. These data reinforce the role of EVOO as a potential adjuvant candidate for future treatments of IL-1β-associated inflammatory diseases. In fact, EVOO was used in patients with gingivitis to manage inflammation, promoting reductions in gingival bleeding and supragingival biofilm [52].

Today, the importance of the human microbiome and its impact on human health has become increasingly evident, and studies are being conducted to deepen the understanding of the microbiome’s relevance, opening the door to potential therapeutic and diagnostic applications [53]. The largest concentration of the human microbiome is found in the gut, and the most predominant phyla in the healthy gut are *Bacillota* and *Bacteroidota*, followed by *Actinobacteria*, *Proteobacteria*, and *Verrucomicrobia* [49]. However, oral microbiota is the second-largest microbiome in the human body and is physically connected with the gut since the oral cavity is the beginning of the digestive system. For that reason, emerging evidence has shown important connections between gut and oral microbiota. Dysfunctional barriers can allow for oral microbes to translocate to the intestinal mucosa, affecting inflammation and immune responses; on the other hand, gut-to-oral microbial transmission also occurs [52]. This gut–oral axis of the two microbiomes may contribute to the pathological processes of many oral and systemic diseases, including diabetes, rheumatoid arthritis, IBD, and colorectal cancer, among others [31,54].

Although some studies have referenced that polyphenol-rich EVOO has a beneficial effect on the gut microbiota, acting as a prebiotic and promoting the growth of beneficial bacteria such as *Lactobacillus* and *Bifidobacterium* [55,56], there are few studies that show the modulation effect of EVOO in the abundance of *Bacillota* and *Bacteroidota,* and none referred to the effect of EVOO on the oral microbiome. In a recent study performed in a mouse model, the effects of three different high-fat diets (sunflower, coconut, and EVOO) on gut microbiota were evaluated [57]. The results showed that sunflower and coconut diets generated a pro-inflammatory intestinal microenvironment, with an increase in inflammatory bacteria. In contrast, the EVOO diet produced an anti-inflammatory microenvironment characterised by the decreased *Enterococcus*, *Staphylococcus*, *Neisseria*, and *Pseudomonas* spp. abundance, which increased the *Bacillota*/*Bacteroidota* ratio. This ratio is a critical marker of the health of both the gut and oral microbiomes influencing microbial balance, immune function, and the development of chronic diseases such as obesity [58], inflammatory bowel disease [59], and cardiovascular diseases [60]. It has been reported that an increase in the *Bacillota*/*Bacteroidota* ratio is usually observed in obese individuals, while a decrease is characteristic of patients with IBD [61], indicating that this ratio is essential to maintaining homeostasis and changes to it may contribute to the development of several diseases. Therefore, *Bacillota*/*Bacteroidota* modulation may impact systemic inflammation and interventions targeting the *Bacillota*/*Bacteroidota* balance through diet, probiotics, and oral care, which are promising strategies for reducing inflammation and improving overall health [61]. Indeed, several studies have demonstrated that the EVOO phenolic compounds increase Bacteroidetes and/or reduce the *Bacillota*/*Bacteroidota* ratio, which have both been related to atheroprotection; moreover, these compounds, which are present in our EVOO, also increase certain beneficial bacteria and gut bacteria diversity, which can be therapeutic for lipid-immune disorders and obesity [56]. Also, the antimicrobial effect of EVOO’s bioactive compounds such as polyphenols can also inhibit the growth of harmful bacteria and facilitate the expansion of beneficial bacteria [62]. These results are in line with those presented in this study, showing EVOO’s modulation effect on gut and oral microbiota, with the increase of *Bacteroidota* in both microbial ecosystems and the increase of *Bacillota* in the oral microbiome, both phyla being associated with health, conferring a prebiotic effect to EVOO. Moreover, it is becoming evident that the microbiome is also significantly altered in many chronic inflammatory diseases, such as IBD, with pro-inflammatory cytokines like the IL-1 family having an important role in shaping the composition of the microbiome at barrier sites [56]. For instance, IL-1β can induce an increase in intestinal barrier permeability, allowing for the permeation of luminal antigens, causing intestinal inflammation. In the case of the oral cavity, the correlation of oral microbiome dysbiosis with oral inflammatory diseases, such as periodontitis, is also well established [63,64]. This oral pathology is highly characterised by microbiome dysbiosis, and IL-1β is upregulated by *Porphyromonas gingivalis*, a periodontopathogen bacteria [31,64]. The results reported herein showed that EVOO can have a critical role in regulating microbiome dysbiosis-mediated inflammation by reducing the pro-inflammatory IL-1β.

Although it is recognised that the sample size could be larger, the data reflect significant changes in the analysed markers. Future research should incorporate for both stool and saliva samples a more in-depth microbiome analysis through high-throughput sequencing methods, specifically 16S rRNA gene sequencing.

## 5. Conclusions

A high-polyphenol content EVOO was extensively studied, mainly because of its beneficial health effects in decreasing the risk of cardiovascular disease and modulating inflammatory markers and glycemia regulation. The northern Portuguese polyphenol EVOO used in this study showed significant positive effects on health, namely in decreasing HbA1c and LDL-C, as well as modulating inflammation by decreasing inflammatory biomarkers, such as the pro-inflammatory interleukin IL-1β, alongside significant changes in microbiota, which was independent of an individual’s diet. The increase of *Bacteroidota* found in both ecological niches and the increase of *Bacillota* found in the oral microbiota, both phyla being associated with health, confer a prebiotic effect to EVOO.

## Figures and Tables

**Table 1:** Participants’ clinical characterisation (adapted from Correia et al., 2023) [5].

Variables and Collected Data	Total n = 33 (8 Men, 25 Women)		*p*
**Age**	33.5 ± 11.2 (19–55)		
**Smoking habits**	Participants did not smoke		
**Chronic/inflammatory diseases**	No chronic/inflammatory diseases		
**IPAQ score**	**Prior to EVOO intake**	**After EVOO intake**	
Sedentary + irregular activity (%)	8 (24.3%)	10 (30.3%)	ns
Active + very active (%)	25 (75.7%)	23 (69.7%)	ns
**Analytical Data**			
HbA1c (%)	5.12 ± 0.32 (4.6–5.8)	4.93 ± 0.24 (4.5–5.6)	0.000 *
Total cholesterol (mg/dL)	168.22 ± 30.93 (79–200)	162.79 ± 32.30 (84–213)	0.208
HDL (mg/dL)	62.91 ± 25.70 (34–184)	62.23 ± 24.53 (39–179)	0.281
LDL (mg/dL)	92.63 ± 24.53 (34–127)	83.23 ± 27.63 (43–138)	0.024 *
CRP (mg/dL)	0.65 ± 1.37 (0.3–7.7)	0.36 ± 0.20 (0.3–1.17)	0.119
**Dietary Inflammation contribution**			
** DII, mean	2.178 ± 0.97 (−0.22–3.36)	1.93 ± 1.21 (−2.01–3.32)	0.335
**Anthropometry**			
BMI	23.65 ± 3.19 (18.5–29.9)	23.71 ± 2.97 (18.5–29.9)	0.331

Values are expressed in number of volunteers (n = 33) and percentage or as mean values ± SD (min–max) accordingly; ns—difference was not significant; * Student’s *t*-test—significant *p*-value, (*p* < 0.05); ** 24 h recall data referring to a sample of n = 32, as one participant had missing values.

## Data Availability

The original contributions presented in this study are included in the article. Further inquiries can be directed to the corresponding author.
